# Aminopyridine treatment for an adult patient of developmental and epileptic encephalopathy with a gain-of-function mutation in KCNA2

**DOI:** 10.1016/j.gendis.2025.101798

**Published:** 2025-08-07

**Authors:** Lehong Gao, Yu Jia, Yingxue Yang, Jia Chen, Liankun Ren

**Affiliations:** a Department of Neurology, Xuanwu Hospital, Capital Medical University, Beijing 100053, China; b Clinical Research Center of Epilepsy, Xuanwu Hospital, Capital Medical University, Beijing 100053, China; c National Center for Neurological Disorders, Beijing 100053, China; d Chinese Institute for Brain Research, Beijing 102206, China

Developmental and epileptic encephalopathies (DEEs) are a phenotypically and genetically heterogeneous group of severe epilepsy accompanied by intellectual disability. Mutations in the *KCNA2* gene have been identified as a potential cause of DEE.^1^ The *KCNA2* gene encodes the Kv1.2 channel, which plays a critical role in the initiation and propagation of action potentials, thereby influencing firing patterns and regulating neuronal excitability.^2^ *KCNA2*-DEE has a broad spectrum of diseases, including drug-resistant seizures, cognitive impairment, and ataxia. Encouragingly, treatments for *KCNA2*-DEE have evolved from traditional anti-epileptic seizure therapies to gene-targeted strategies. 4-Aminopyridine (4-AP), a non-specific inhibitor of voltage-gated potassium channels, was reported to be effective in treating *KCNA2*-DEE in 2021.^3^ To date, 9 of 11 patients have benefited from treatment with 4-AP.^3^ However, further clinical evidence is necessary to determine the generalizability of these effects, particularly in adult patients with a long course beginning in childhood. Here, we report an adult patient with a 30-year history of epileptic encephalopathy beginning in infancy, carrying the *KCNA2* gain-of-function variant p.(Arg297Gln), who benefited from treatment with 4-AP.

The patient was a 31-year-old male born at full term via spontaneous vaginal delivery to healthy, non-consanguineous parents (Fig. 1). He achieved early developmental milestones appropriately, beginning to speak at 10 months and walk at 18 months. He denied a history of intracranial infection or febrile seizures. Seizure onset occurred at 15 months, characterized by focal to bilateral tonic‒clonic seizures, with a frequency of 7–8 times per year. Following disease onset, both cognitive and motor development lagged behind that of his peers. The cluster spasms started at the age of 6, occurring 2–3 times daily. Concurrently, he exhibited cognitive regression, unsteady gait, frequent falls, and slow and incoherent speech. He dropped out of school at the age of 8. The patient had been treated with multiple anti-epileptic drugs, and at the age of 20, the regimen was modified to oxcarbazepine (600 mg bid), valproate (250 mg bid), and clonazepam (0.5 mg qn). The cluster spasms resolved, and the frequency of seizures decreased. However, the patient experienced atypical absence seizures with eyelid myoclonus at the age of 25 and experienced continuous myoclonic seizures at the age of 27, each lasting 1–2 min and occurring 2–3 times weekly. Prior to hospitalization, the patient could dress and eat independently but persisted frequent falls. He required assistance with complicated movements.

**Figure 1:**
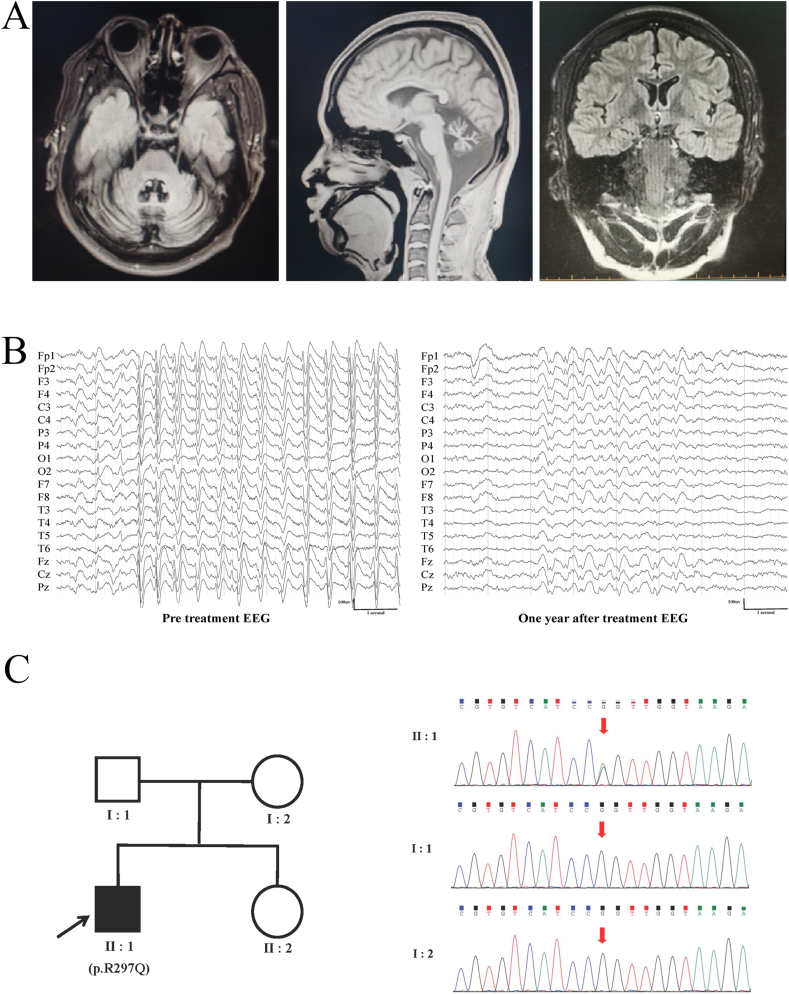
Neuroimaging, EEG, and genetic findings in a KCNA2-related encephalopathy. **(A)** Brain MRI features of the patient with the KCNA2 mutation. Severe cerebellar and moderate pontine atrophy on axial and sagittal T1-weighted images. **(B)** The EEG results of the patient before and after 4-AP treatment. The interictal EEG before treatment showed generalized medium-to-high amplitude 1.5–2.5 Hz spike-and-slow wave/multi-spike-and-slow wave rhythmic discharges. EEG after treatment showed that epileptic discharges were less stereotypical and of shorter duration. **(C)** The WES analysis results of the patient. Familial pedigrees and Sanger sequencing confirmation of *KCNA2* variants. The *de novo* c.890G > A mutation was identified in the proband.

The patient exhibited cognitive decline, hypotonia, hyperactive tendon reflexes in both lower limbs, and cerebellar ataxia. Her bilateral Babinski's sign was positive. Routine blood tests, blood biochemistry, thyroid function, blood ammonia levels, and urinary organic acid screening were all within normal limits. The electrocardiogram was normal with a corrected QT interval of 388 ms. The MoCA score was 4. Brain MRI revealed atrophy of the pontine, left hippocampus and cerebellum (Fig. 1). Interictal EEG showed generalized low-to-medium amplitude 4–6 Hz slow-wave activity, along with generalized medium-to-high amplitude 1.5–2.5 Hz spike-and-slow-wave/multi-spike slow-wave rhythmic discharges. Atypical absence seizure onset with myoclonus was recorded. Ictal EEG showed a generalized medium-to-high amplitude 4–5 Hz slow spike-and-slow-wave rhythm (Fig. 1). Genomic DNA from the proband and his parents was extracted from peripheral blood using standard protocols. DNA libraries were constructed using a SureSelect All Exon 6 (Agilent) kit. Whole-exome sequencing identified that the patient carried a *de novo* missense variant in *KCNA2* c.890G > A; (p.Arg297Gln), which was confirmed by Sanger sequencing (Fig. 1). According to criteria from the consensus recommendation of the American College of Medical Genetics and Genomics and the Association for Molecular Pathology, this variant was categorized as pathogenic (see materials and methods in supplementary data for more details).

Therefore, the patient was diagnosed with *KCNA2-*DEE based on the clinical manifestations and the pathogenic gene variant. The original anti-epileptic drug regimen was maintained, and 4-AP was initiated at a dose of 5 mg once daily. Adverse effects were closely monitored, and serial electrocardiograms revealed no QT interval prolongation. After two weeks at the initial dose, the 4-AP dosage was increased to 10 mg. One month after treatment initiation, the patient achieved seizure freedom. The patient's family expressed satisfaction with the therapeutic response but remained concerned about potential side effects, opting to maintain the 10 mg dosage long-term. The 10 mg dosage was continued, and notable improvements in speech function were observed after three months of treatment. The tolerability of 4-AP was excellent, with no significant adverse effects recorded in our patient. At the 1-year follow-up, the patient remained seizure-free, exhibited improved language fluency, reduced ataxia, and an absence of falls. Follow-up EEG was performed regularly during treatment. Epileptiform discharges were quantified by three experienced EEG technicians, with each slow spike-and-wave complex counted as one epileptiform discharge. Although the 1-year follow-up EEG still revealed generalized medium-to-high amplitude, 1.5–2.5 Hz spike-and-slow-wave discharges (Fig. 1), these discharges were less stereotypical and of shorter duration. Moreover, a 24-h EEG recorded 96 epileptiform discharges, a substantial reduction from the 1548 discharges recorded prior to treatment. The patient continues on the current treatment regimen, and follow-up evaluations are ongoing to assess the long-term efficacy of 4-AP in *KCNA2*-DEE.

In recent years, advances in precision medicine and the development of potassium ion channel-targeted therapies for *KCNA2*-DEE have shown great promise in both clinical trials and applications. The *KCNA2* variant (p.Arg297Gln) is classified as a GOF mutation of Kv1.2. Known GOF mutations in *KCNA2*, including p.Leu298Phe, p.Arg297Gln, and p.Glu157Lys, have been shown i*n vitro* to be antagonized by 4-AP, which reduces current amplitudes, induces a negative shift in steady-state activation, and increases the firing rate of transfected neurons.^3^ In this study, we report an adult patient with a 30-year history of epileptic encephalopathy who harbored the *KCNA2* p.(Arg297Gln) mutation. The patient was followed for one year and remained seizure-free, with marked improvements in speech and ataxia.

Four patients with the *KCNA2* mutation (p.Arg297Gln) were treated with 4-AP.^3^ Treatment was initiated between the ages of 3.5 and 37 years and led to improvements in seizures, ataxia, and cognition. The most critical factor associated with optimal treatment response was the timing of initiation, with greater efficacy observed when 4-AP was administered at preschool and school ages. The reported dosages ranged from 20 to 55 mg/day, with two adults receiving 30–40 mg/day. In our case, he began 4-AP treatment at the age of 31. Despite receiving a lower dosage than previously reported, he exhibited a favorable clinical response and achieved seizure freedom within three months of treatment. This case provides an evidence for the good efficacy of 4-AP treatment in adult patients with *KCNA2*-DEE. Notably, since Kv1.2 and other potassium channels are expressed in the human ventricular myocardium, 4-AP has been shown to prolong action potentials in canine ventricular cell models.^4^ Nevertheless, a low dose of 4-AP was well tolerated in our patient without adverse effects such as dizziness, paresthesia, nausea, or cardiac side effects.^1^^,^^5^ Furthermore, higher doses of 4-AP may also be considered safe for patients with Kv1.2 GOF mutations, as 4-AP may reduce the activity of Kv1 channel complexes to physiological levels without exceeding suppression as might occur in individuals with normal Kv1 channel activity.^3^

In summary, we report 4-AP treatment for an adult case with *KCNA2*-DEE. Despite the long duration of DEE, the patient's symptoms improved following a short course of low-dose, targeted treatment with 4-AP. Further follow-up is required to evaluate the long-term treatment outcomes of 4-AP treatment for *KCNA2-*DEE.

## CRediT authorship contribution statement

**Lehong Gao:** Writing – review & editing, Validation, Supervision, Funding acquisition, Conceptualization. **Yu Jia:** Writing – original draft, Methodology, Investigation, Conceptualization. **Yingxue Yang:** Methodology, Investigation, Formal analysis. **Jia Chen:** Methodology, Investigation. **Liankun Ren:** Writing – review & editing, Methodology, Conceptualization.

## Ethics declaration

Written informed consent was obtained from all study participants. All procedures were approved by the ethics committee of Xuanwu Hospital (No. [2024]393-001).

## Funding

This study was supported by the 10.13039/501100012166National Key Research and Development Program of China (No. 2022YFC2503802), the 10.13039/501100012166National Key Research and Development Program of China (No. 2022YFC2503805), and the 10.13039/501100001809National Natural Science Foundation of China (Nos. 82401705, 82371461).

## Conflict of interests

The authors declared no competing interests.
