# Supplementary material for: Aminopyridine treatment for an adult patient of developmental and epileptic encephalopathy with a gain-of-function mutation in KCNA2
Source: Genes Dis. 2025 Aug 7;13(4):101798. doi: 10.1016/j.gendis.2025.101798 (PMC13011020; doi:10.1016/j.gendis.2025.101798)
Supplement: Multimedia component 1 [file mmc1.docx]

**Detailed Materials and Methods**

**1.patient identification and Clinical examination**

The patient was enrolled from Department of Neurology, Xuanwu Hospital, Capital Medical University, Beijing. The detailed clinical information, including demographics, laboratory test, EEG and MRI results (MAGNETOM Skyra 3.0T), etc. were collected. The patient underwent EEG examinations before and after 4-AP treatment. Epileptiform discharges were quantified by three experienced EEG technicians, with each slow spike-and-wave complex counted as one epileptiform discharge. Written informed consent was obtained from the study participant. All procedures were approved by the ethics committee of Xuanwu Hospital (No. [2024]393-001).

**2.Whole-exome sequencing and genetic analysis**

The proband’ and his parents’ genomic DNAs were extracted from peripheral blood using standard methods. The DNA library was constructed using a SureSelect All Exon 6 (Agilent) kit. Whole exome sequencing (WES) were performed on the NovaSeq6000 platform (IlluminaInc., San Diego, CA, United States). Paired-end reads were aligned to the reference humang enome sequence (GRCh37/hg19) using BWA algorithm.^1^ The bioinformatic analysis was performed by GATK, samtools and bcftools packages. Mean sequencing depth of 128× and 99.7% of targeted regions covered at 20×depth were obtained. Chromosomal position, frequencies, and other relevant information of variants was annotated using GnomAD database (https://gnomad.broadinstitute.org/), 1000 Genome Project database (http://www.1000genomes.org), dbSNP version 147 (http://www.ncbi.nlm.nih.gov/projects/SNP), and the Exome Aggregation Consortium (ExAC) (http://exac.broadinstitute.org/) database. WES analysis identified a *de novo* missense variant in KCNA2 c.890G>A;(p.Arg297Gln), as shown in Figure 1C. In accordance with the recommendation of the American College of Medical Genetics 5and the Association for Molecular Pathology (ACMG / AMP),^2^ the variant was categorized as a “pathogenic (P) variant”. The following detailed evidences were included: PM2_PP +PP3_S + PS3_PP+PS2+PS4.

**Supplementary Table 1.** 4-AP treatment for patients with KCNA2 mutation (p.(Arg297Gln)) from publications.^3^

| **Case No.** | **Gender** | **Age at**  **onset**  **(months)** | **Age at start of**  **treatment**  **(years.months)** | **Treatment duration**  **(months)** | **Max.**  **dosage**  **(mg/d)** | **Effect on**  **seizures** | **Effect**  **on**  **ataxia** | **Effect on**  **cognition**  **and speech** |
| --- | --- | --- | --- | --- | --- | --- | --- | --- |
| 1 | Female | 1 | 3.5 | 19 | 20 | ++ | ++ | ++ |
| 2 | Female | 10 | 4.10 | 73 | 55 | Seizure free | ++ | + |
| 3 | Male | 5 | 26 | 75 | 40 | Seizure free | + | + |
| 4 | Female | 10 | 37 | 65 | 30 | + | + | + |

**Supplementary references**

1.Li H, Durbin R. Fast and accurate short read alignment with Burrows-Wheeler transform. *Bioinformatics*. 2009; 25(14): 1754-1760. doi:10.1093/bioinformatics/btp324

2.Richards S, Aziz N, Bale S, et al. Standards and guidelines for the interpretation of sequence variants: a joint consensus recommendation of the American College of Medical Genetics and Genomics and the Association for Molecular Pathology. *Genet Med*. 2015;17(5):405-424. doi:10.1038/gim.2015.30

3.Hedrich UBS, Lauxmann S, Wolff M, et al. 4-Aminopyridine is a promising treatment option for patients with gain-of-function KCNA2-encephalopathy. *Sci Transl Med*. 2021;13(609):eaaz4957. doi:10.1126/scitranslmed.aaz4957
